# Ending COVID-19: progress and gaps in research—highlights of the July 2020 GloPID-R COVID-19 Research Synergies Meetings

**DOI:** 10.1186/s12916-020-01807-3

**Published:** 2020-10-29

**Authors:** The COVID-19 Research GloPID-R Synergies Meeting Working Group, Geneviève Boily-Larouche, Gail Carson, Josie Golding, Evelyn Depoortere, João Rangel de Almeida, Richard Vaux, Giuseppe Paparella, Danielle Vitali, Deborah Khursigara, Claire Madelaine, A. Morgan Lay, Barbara Kerstiëns, Yazdan Yazdanpanah, Charu Kaushic, Meeting Co-chairs, Anita Zaidi, Melanie Saville, Debra Yeskey, Glenda Gray, Valdiléa Veloso, Marion Koopmans, David Fisman, Kristy Crooks, Kenneth Camargo

**Affiliations:** 1 Canadian Institutes of Health Research – Institute of Infection and Immunity, Hamilton, Canada; 2 ISARIC, Oxford University, Oxford, UK; 3 Wellcome, London, UK; 4 European Commission, Brussels, Belgium; 5 Merieux Foundation, GloPID-R Secretariat, Lyon, France; 6 INSERM, Paris, France; 7 Canadian Institutes of Health Research – Institute of Population and Public Health, Toronto, Canada; 8 Bill and Melinda Gates Foundation, Seattle, USA; 9 CEPI, Washington, D.C., USA; 10 South African Medical Research Council, Cape Town, South Africa; 11 Instituto Nacional de Infectologia Evandro Chagas – Fiocruz, Rio de Janeiro, Brazil; 12 Erasmus MC, Rotterdam, Netherlands; 13 University of Toronto, Toronto, Canada; 14 Menzies School of Health Research Casuarina, Charles Darwin University, Darwin, Northern Territory Australia; 15 Rio de Janeiro State University, Rio de Janeiro, Brazil

**Keywords:** COVID-19, SARS-CoV-2, GloPID-R, COVID-19 vaccines, COVID-19 therapeutics, SARS-CoV-2 transmission, Social sciences

## Background

In mid-July, GloPID-R convened its members, scientists who had received COVID-19 funding, and world leaders in pandemic preparedness and response for a series of COVID-19 Research Synergies Meetings with the purpose of establishing collaboration and identifying knowledge gaps, in order to build a collective path forward to end COVID-19. GloPID-R is an international consortium of 29 funding organisations invested in research related to new or re-emerging infectious diseases [1]. By offering a coordination platform, GloPID-R aims to maximise the use of available resources and streamline research efforts amongst national and global funders. The COVID-19 pandemic has caused unprecedented devastation to the health and economies of countries across the world and has highlighted basic inequalities in society. Understanding the fundamentals of COVID-19 and building on a collaborative approach between researchers and institutions to achieve health equity, universal access and data sharing is critical to reduce the impact of the COVID-19 pandemic.

“Lockdowns, in my view, have been successful in helping to reduce transmission and new infections, but they haven’t changed the fundamentals of this infection. The virus biology, its transmission, how infectious it is, the clinical syndrome it causes, the inequalities it drives through the world, and of course the tertiary consequences in economics and in geopolitics. So, I think we are only very much at the start and we need to be humble about the challenge we face. We do not yet have a clear exit strategy and we need to define one.” J. Farrar, Wellcome

## Ending COVID-19: vaccines

Balancing speed, scale and access.

Presenters highlighted the progress in COVID-19 vaccine development, regulatory considerations, as well as gaps in advancing the demonstration of clinical efficacy and effectiveness and challenges that must be overcome to meet the global demand for equitable access (Table 1). COVID-19 introduced a paradigm shift in vaccine development from a sequential progression in the pipeline to a parallel approach, requiring adaptation of vaccine testing and the development process. The emergence of at-risk manufacturing highlighted the need for built-in flexibility in manufacturing capabilities, along with portfolio approaches to meet global demand. To this end, several initiatives are underway to address global access to COVID-19 vaccines, including COVAX and the WHO technology access pool. Whilst there is sufficient global manufacturing capacity, the need to avoid vaccine nationalism was highlighted to enable a strategic sharing of finite supplies and facilitate equitable global access to COVID-19 vaccines.

**Table 1:** Research gaps identified during the GloPID-R COVID-19 Research Synergies Meetings

Research gaps identified	
**Vaccines**	
o Harmonisation of endpoints and regulatory requirements that will facilitate multinational trials, the evaluation of vaccine candidates and comparisons across studies.	
o SARS-CoV-2 prevalence data that will support the choice of clinical sites and trial designs that can adapt to the evolving pandemic.	
o Define the immune correlates of protection to understand if and how long-term memory can be induced through vaccination.	
o Explore social and behavioural aspects related to vaccines to prepare populations and address vaccine hesitancy for when a vaccine becomes available.	
**Therapeutics**	
o Integrate innovative methods, such as computer-aided drug design in search of effective therapeutics, particularly in the absence of promising strategies out of wet labs.	
o Define the efficacy of combination treatment and gain a better understanding of the interactions between different drugs, and the role of stage of illness, comorbidities and coinfections in treatment effectiveness.	
**Transmission**	
o Increase use of phylogenetic analyses to distinguish sporadic disease importation from amplification and to better understand “superspreader” events.	
o Better investigate the role of children in transmission, in particular the superspreading potential of school settings and the long-term consequences of SARS-CoV-2 infection in children.	
o Understand the role of airflows and aerosol transmission, and why some infected patients do not transmit the virus further.	
o Understand whether transmission from humans to animals might be a concern in future waves/resurgences.	
o Understand SARS-CoV-2 potential circulation in animal populations.	
**Social Sciences Research**	
o Understand social contexts and social rules to better engage communities and achieve control of outbreaks, considering that vaccines may or may not be available, or equitably available between different social groups.	
o Use qualitative and quantitative methods to highlight those individuals and populations who are falling through the cracks, i.e. the most vulnerable, or those presenting cumulative vulnerabilities: the homeless, people who use drugs, inmates, sex workers and migrant workers.	

## Ending COVID-19: therapeutics

Strengthen connection between pre-clinical evidence and clinical trials.

This second session covered the advances in research on therapeutic compounds, clinical trials investigating therapeutic effect and the key remaining questions (Table 1). Repurposing of existing drugs was identified as a valuable avenue for therapeutics, allowing for efficient use of time and resources, and has the additional advantage of building on existing partnerships with industry. Stronger coordination for large-scale clinical trials is needed to avoid fragmentation and to capitalise on international collaboration, with contextual differences being considered, for example, as they relate to standard of care or prevalent co-morbidities. Standardisation of data collection is imperative to allow for comparisons and pooling of data between studies and regions. Inclusive data sharing policies should safeguard data ownership and autonomy [2, 3]. Innovative adaptive trial methods are instrumental to allow testing of multiple compounds and combinations, as well as different levels of data stratification. Communities should be empowered for involvement in clinical trial decision-making structures.

## Preparing for a second wave: understanding transmission and how to stop COVID-19

Multi-disciplinary and holistic approach, with a variety of perspectives including epidemiology, natural sciences, social sciences, aerobiology and architectural design.

The third session focused on understanding current gaps in transmission mechanisms and the measures that might prevent or reduce future waves of infections or resurgences. Several research gaps emerged during this session (Table 1) and all participants agreed that much work is needed to design multidisciplinary outbreak investigation protocols that address key knowledge gaps using state of the art methodologies currently only available in a limited number of laboratories and countries. Such targeted in-depth studies can supplement the data collected through public health reporting and outbreak investigations. Although the generation of evidence and rapid publication has occurred at an accelerated pace, there was agreement that more effort is needed to ensure that robust and comprehensive data is communicated to enable the research community and policy-makers to adequately interpret the relevant findings, which at times has been challenging.

## Social sciences research in COVID-19

Understanding the unequal burden of COVID-19 across social groups.

The Social Science session explored how COVID-19 is shaping the lives of communities and affecting research practices, in four parallel panels: Populational Experiences, Communications and Engagement, Governance and Methodologies. Better ways to implement research findings and redesign research agendas were discussed along with specific pressing research needs (Table 1). The multi-faceted unequal burden of COVID-19 on certain groups was discussed. COVID-19 has affected Indigenous populations in complex ways, introducing three layers of disadvantages: first, these groups have seen their access to health systems’ resources further compromised; second, their reduced political capital has limited their capacity to participate in the allocation of state resources; and third, the current inability to conduct on-site research has prevented social scientists to fully understand the impact of the outbreak amongst these communities, thus creating an additional layer of invisibility and preventing adequate resource mobilisation for supporting these populations.

## Conclusions

Overall, the GloPID-R Synergies Meetings supported a series of collaborative dialogues that led to the identification of crucial knowledge gaps for ending COVID-19. GloPID-R has synergised research efforts and will continue to timely fund and disseminate the critical knowledge needed to inform an exit strategy. A combination of behavioural changes, enhanced public health response, protection of vulnerable populations, accessible diagnostics and interventions and equitable access to effective therapeutics and vaccines, supported by policy research, is the only way forward. Establishing this combination of measures will be contingent on our collective capacity to enable large and coordinated efforts such as multi-centre harmonised clinical trials, and global databases, and support multi-disciplinary research, and knowledge mobilisation to leverage science for timely, sound and transparent decision-making at national and global levels. If even one country is not safe, the rest of the world will not be safe.

“It will take enlightened, brave leadership and concerted action to promote multilateral, cooperative solutions. […] Doing the right thing, doing the thing that is appealing from a humanitarian perspective, is also the efficient thing where ending the pandemic is concerned.” R. Hatchett, CEPI

## Data Availability

Not applicable
